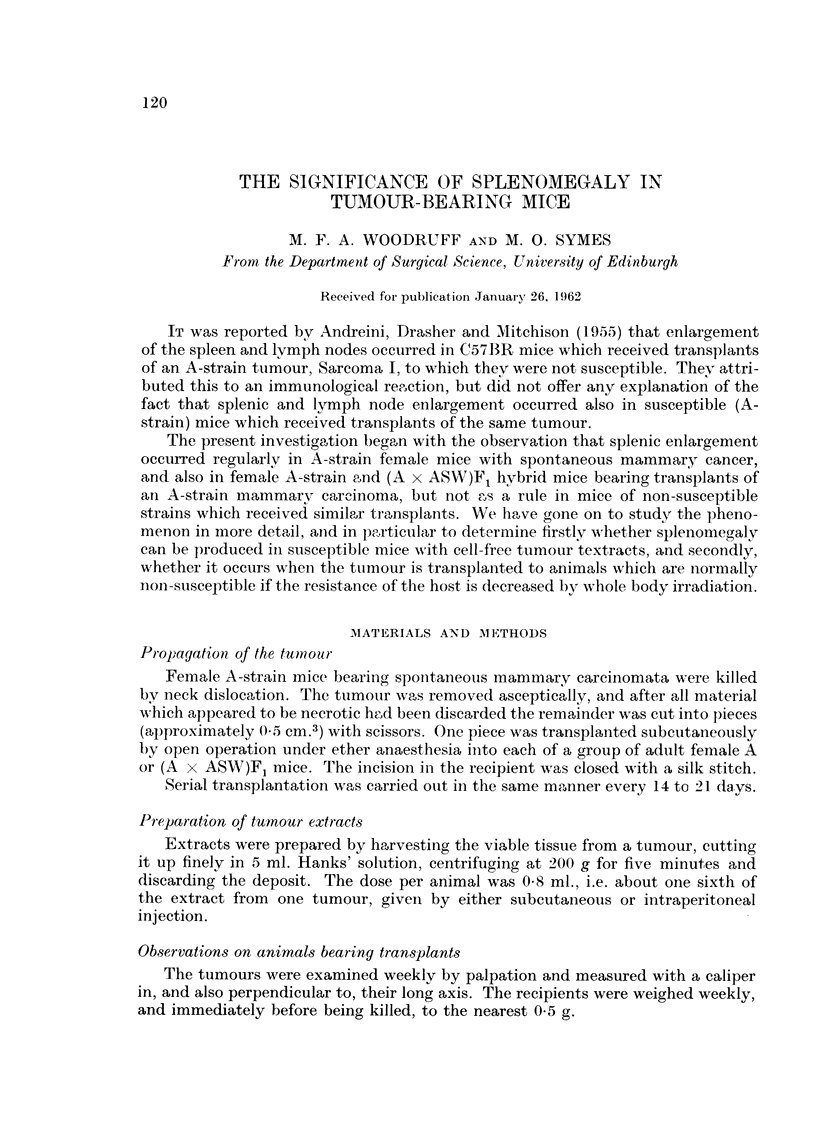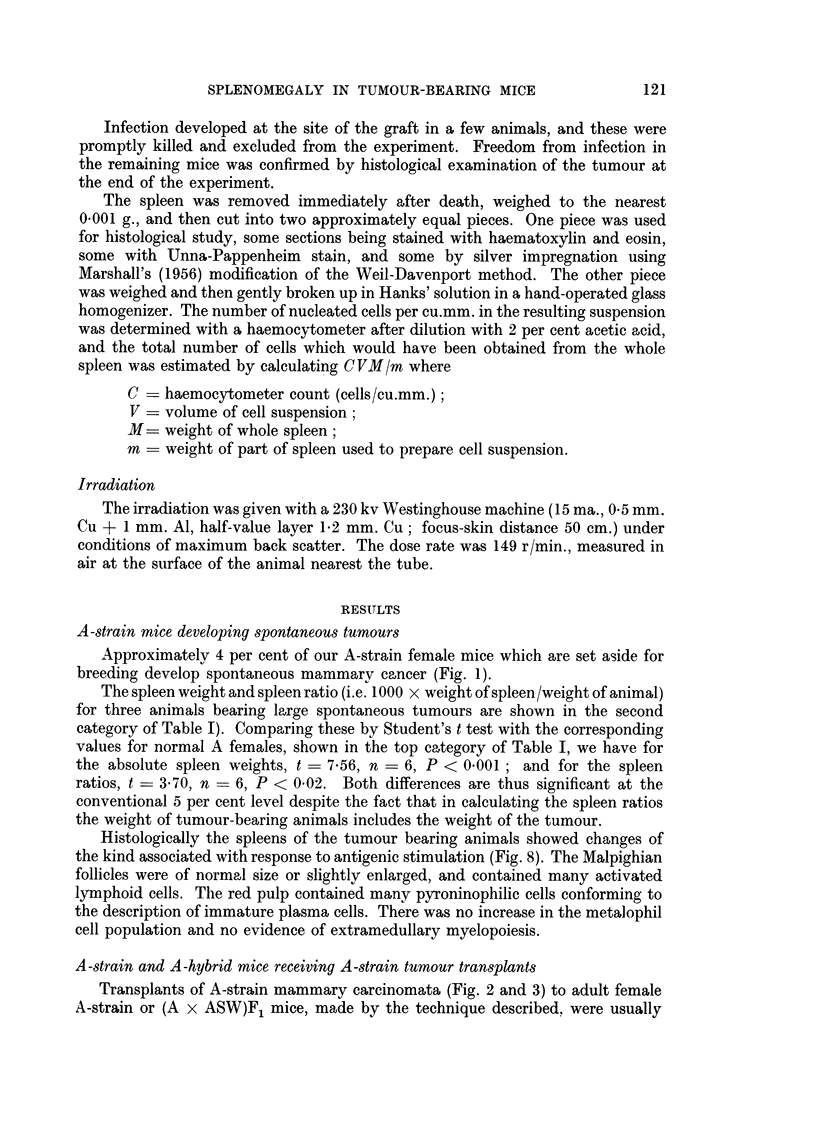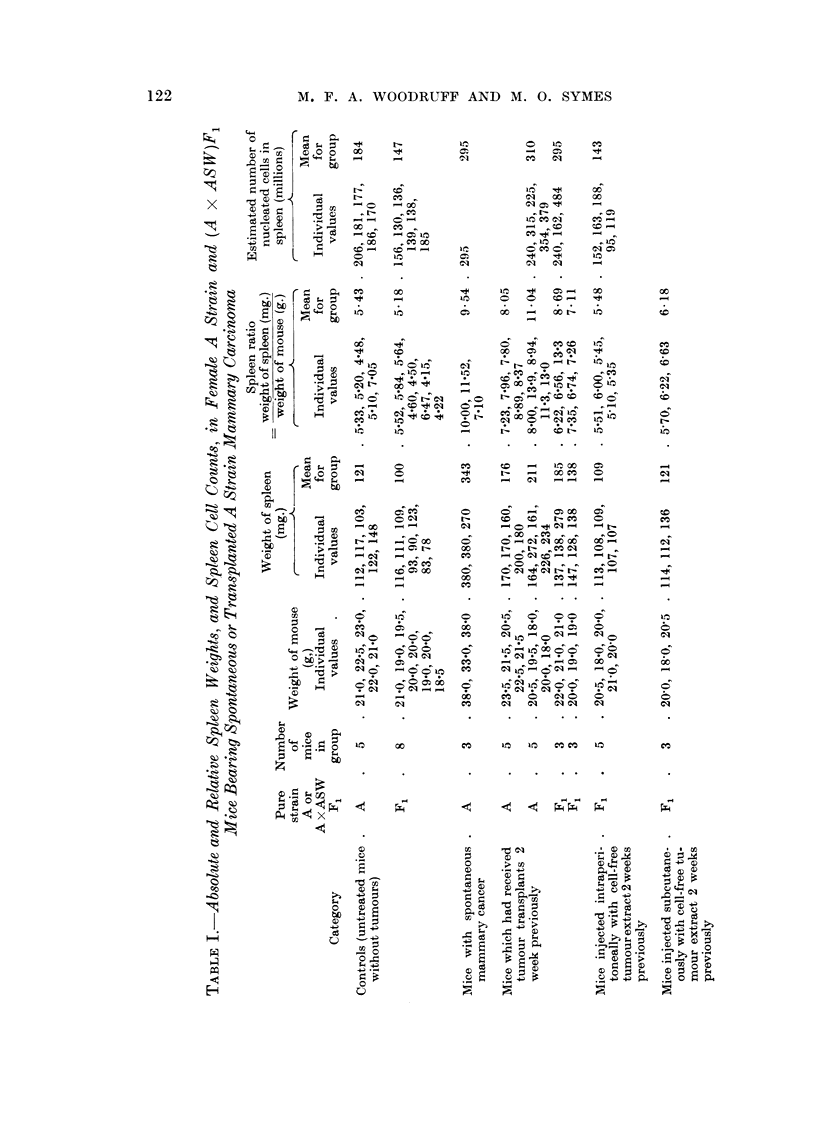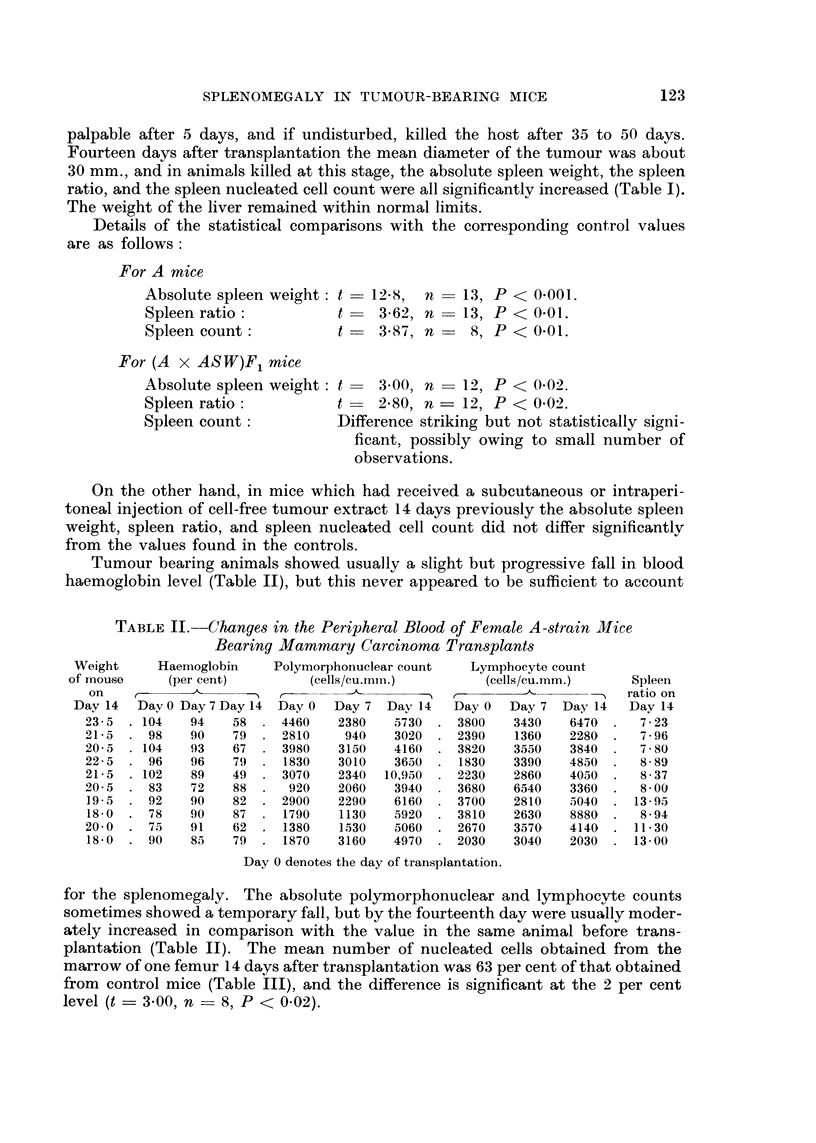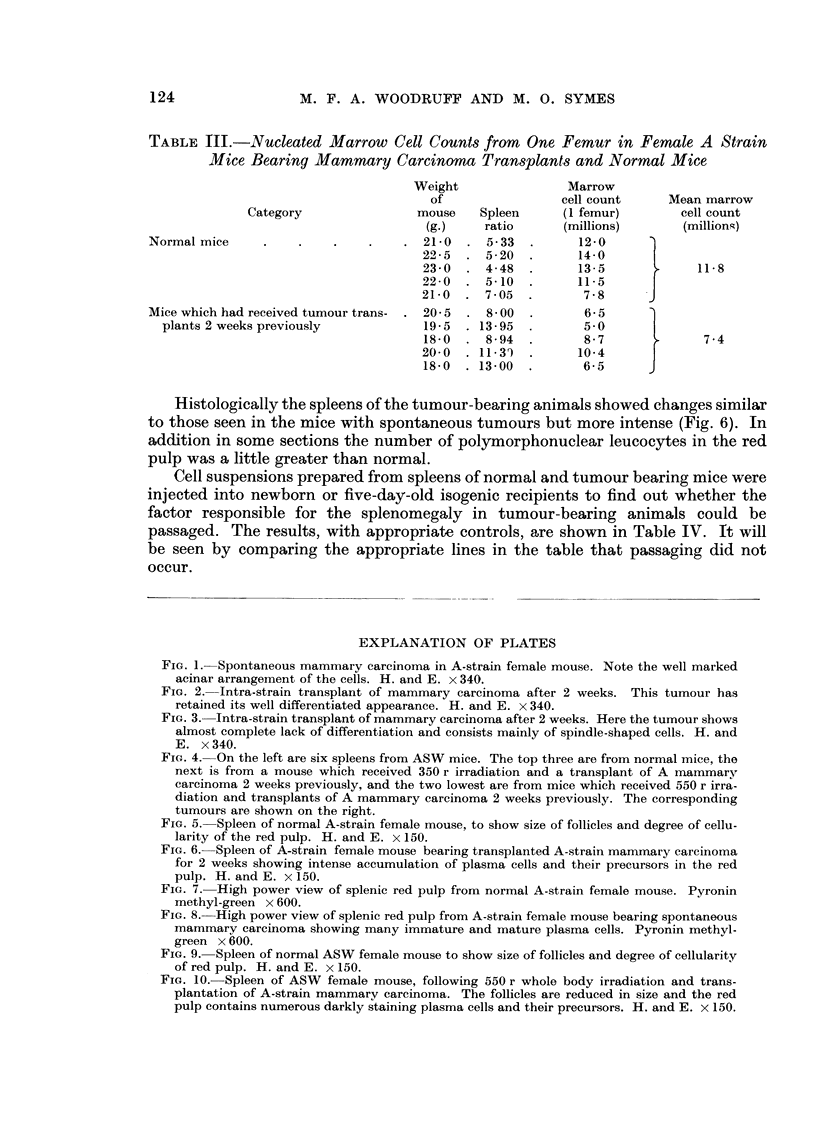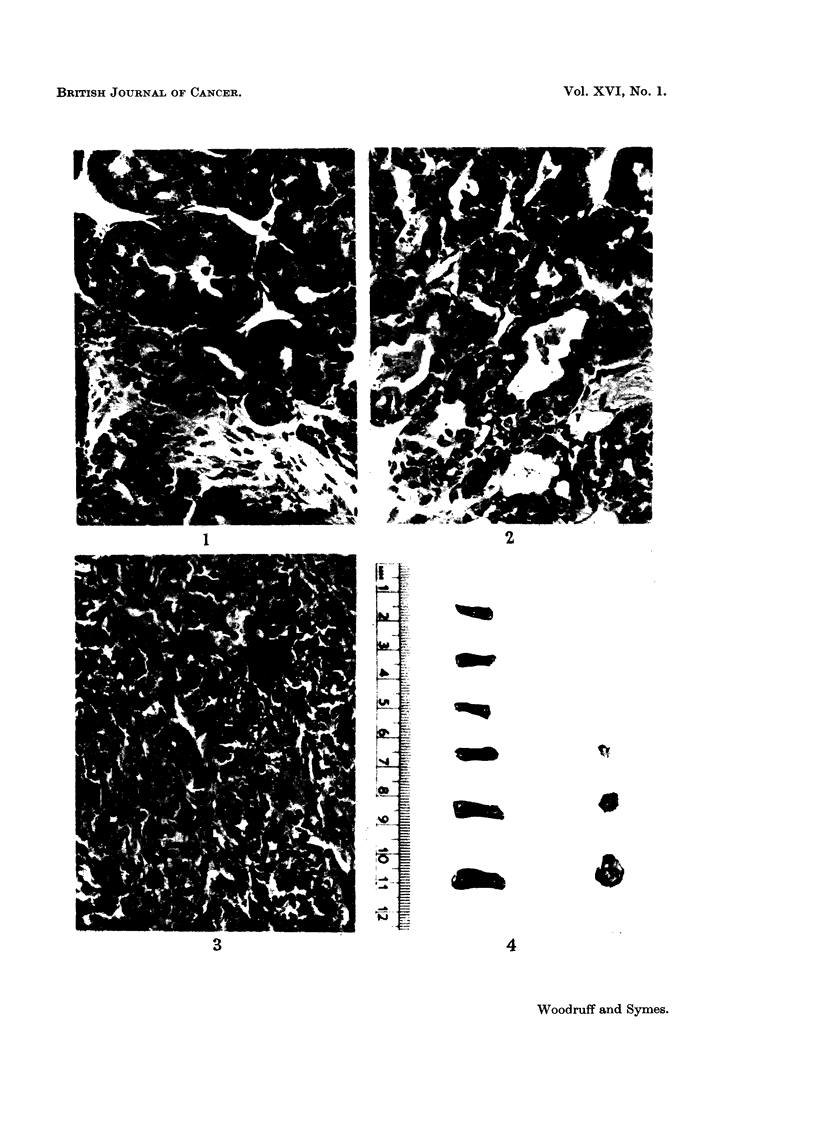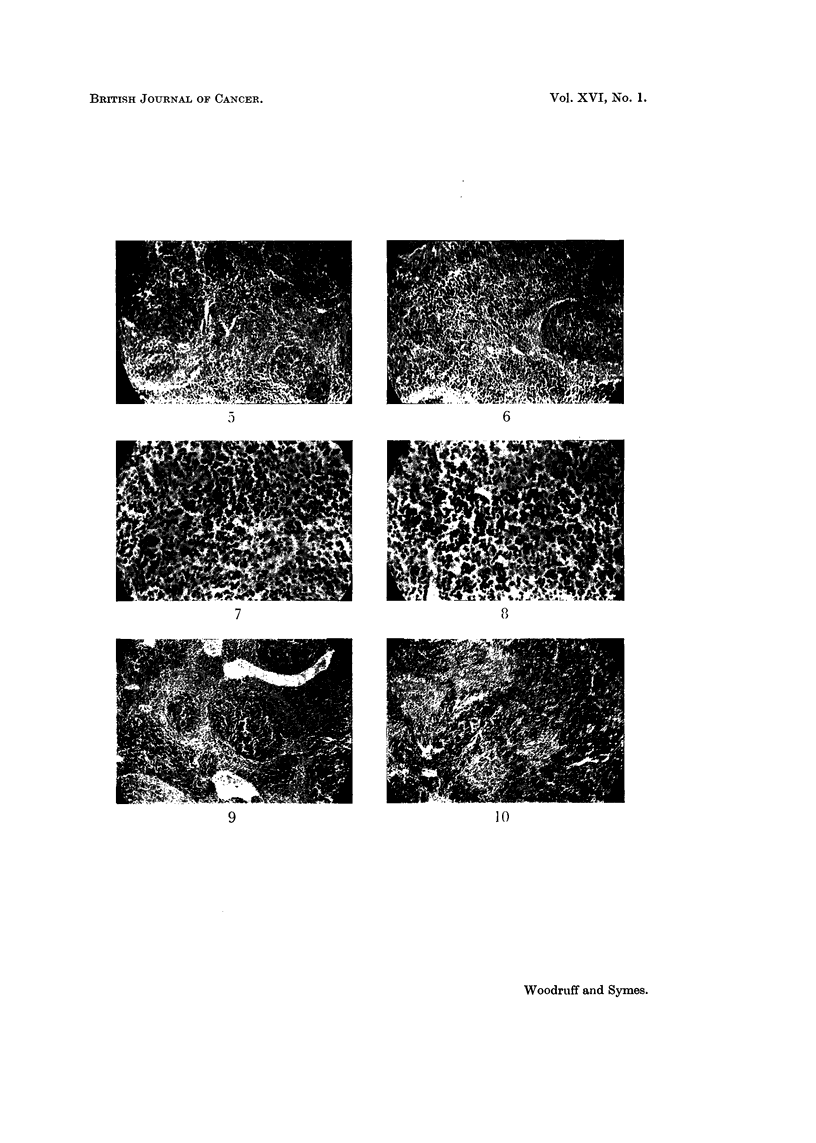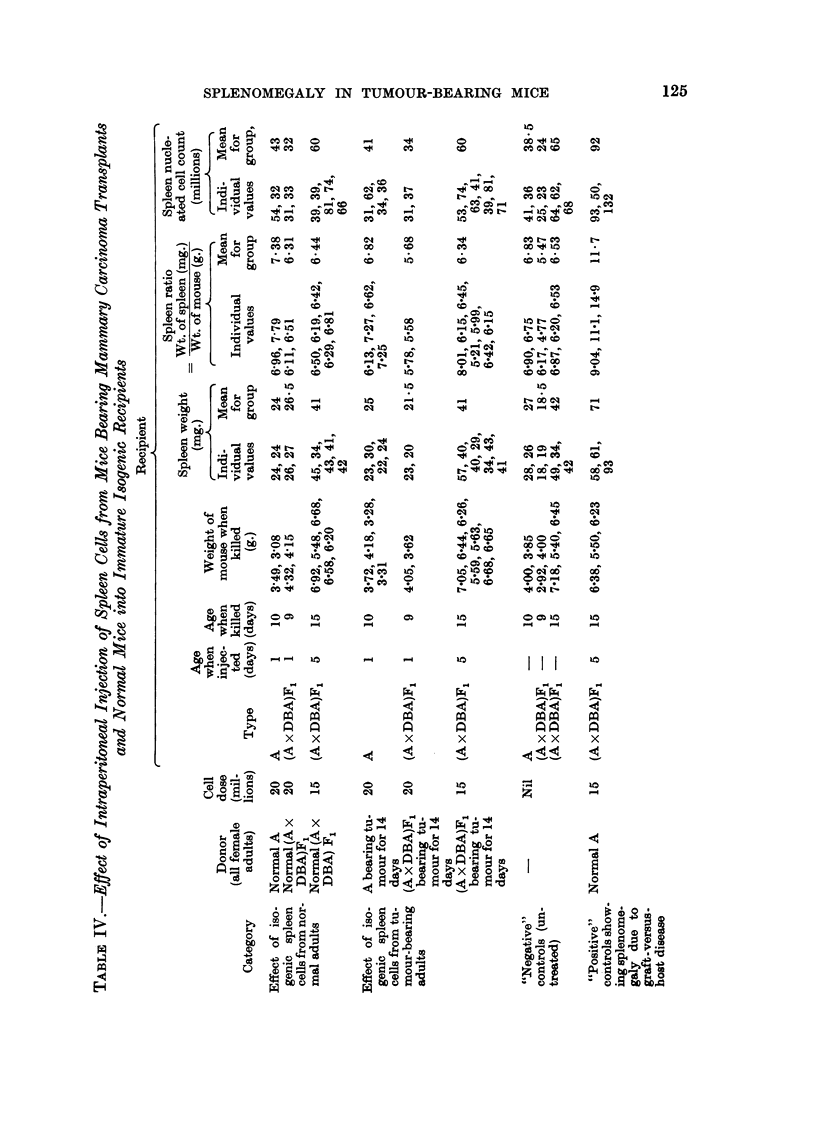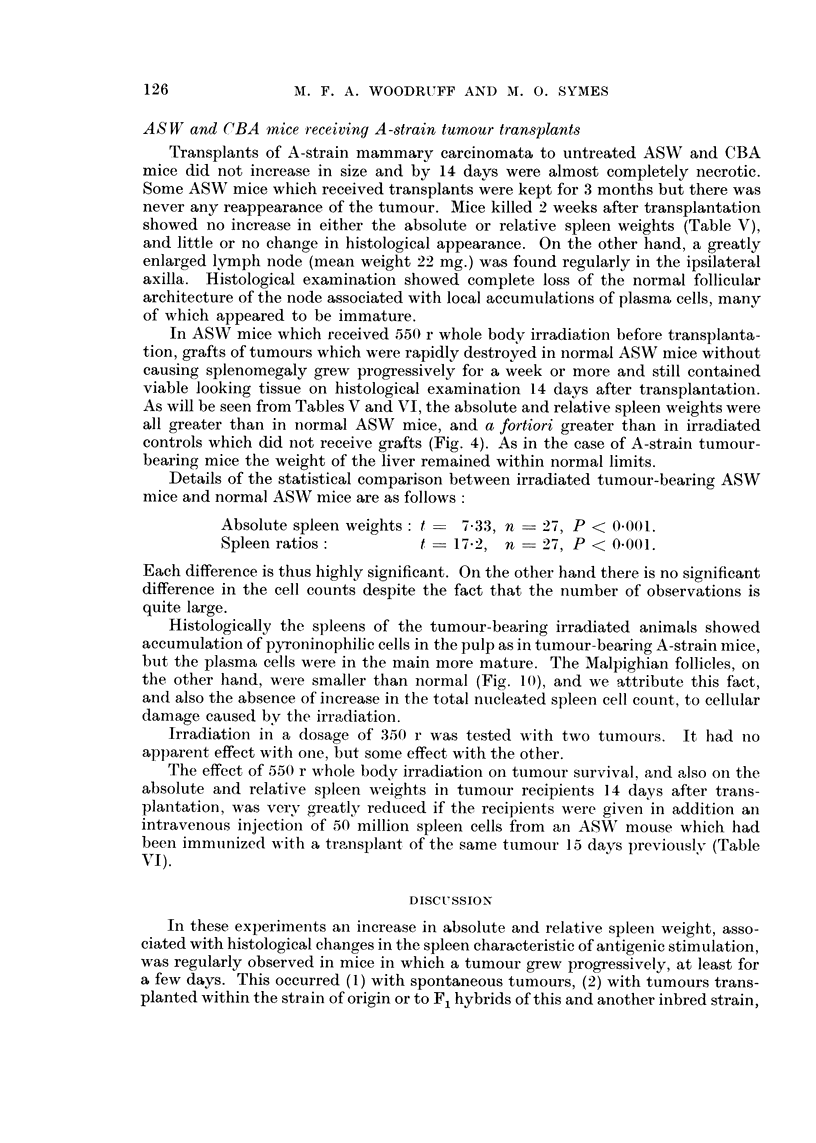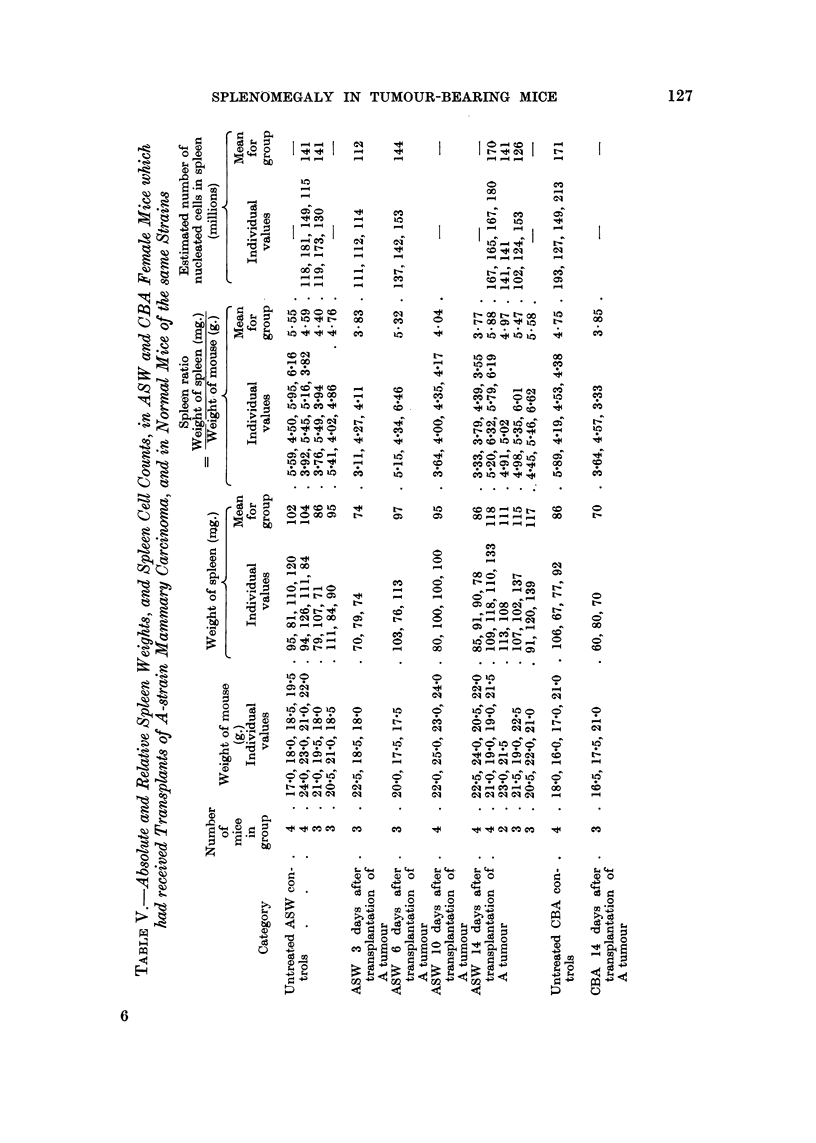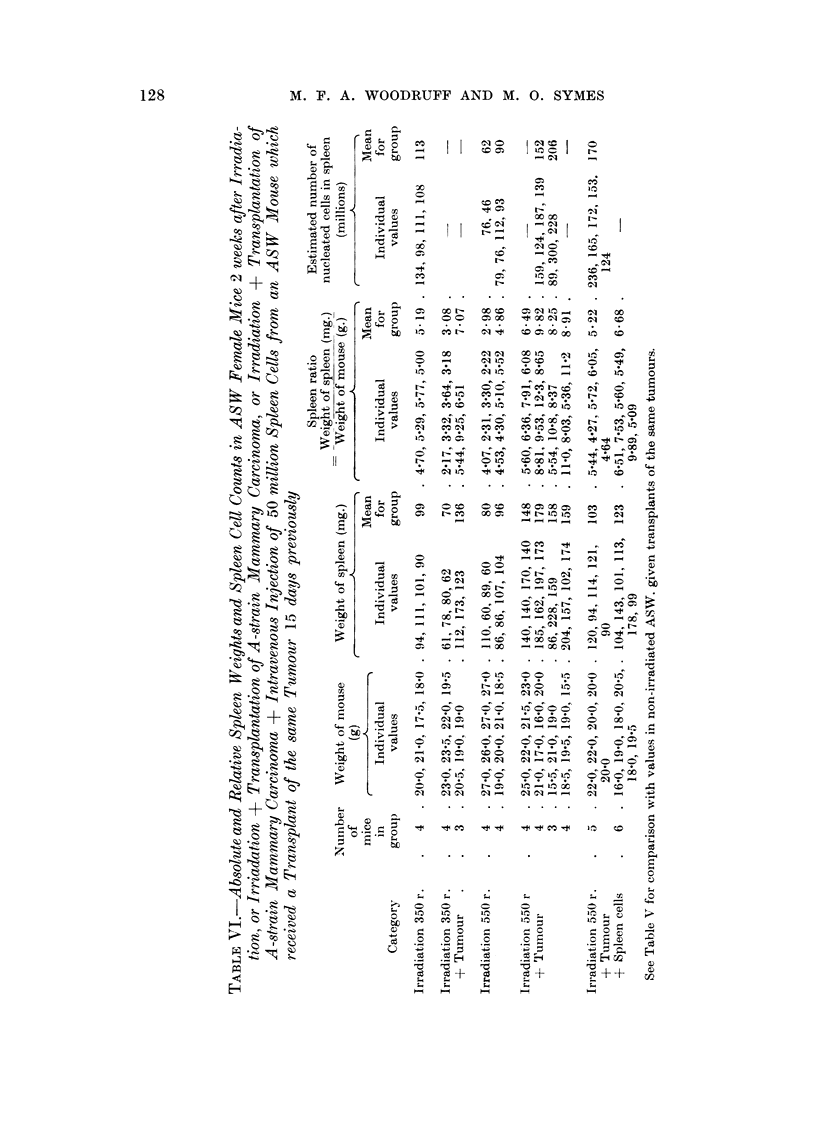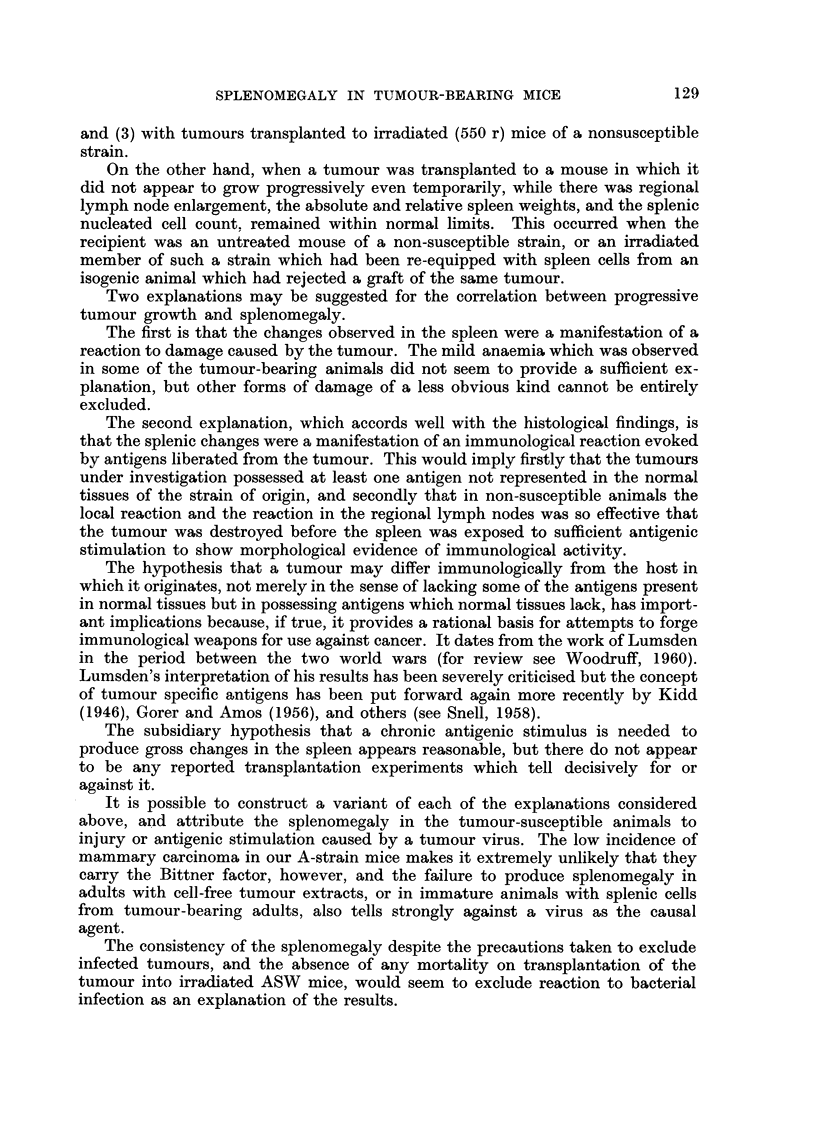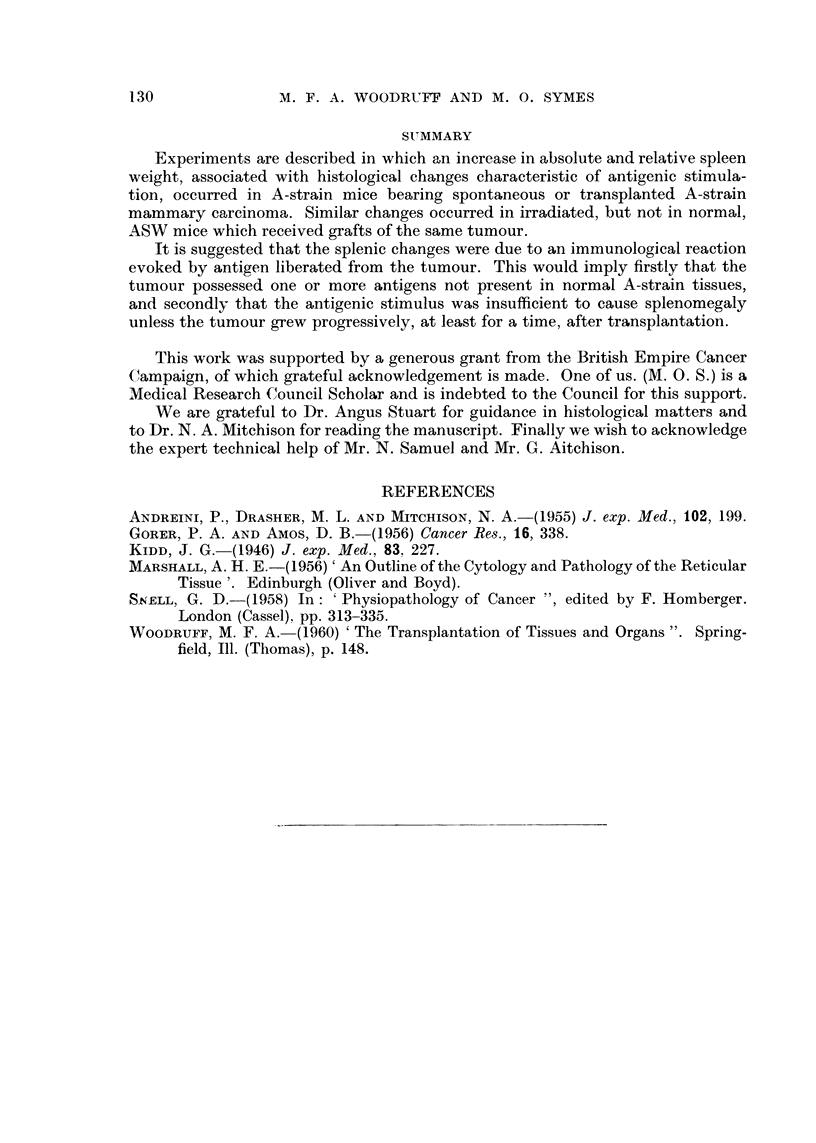# The Significance of Splenomegaly in Tumour-Bearing Mice

**DOI:** 10.1038/bjc.1962.12

**Published:** 1962-03

**Authors:** M. F. A. Woodruff, M. O. Symes

## Abstract

**Images:**


### THE SIGNIFICANCE OF SPLENOMEGALY IN TUMOUR-BEARING MICE

M. F. A. WOODRUFF AND M. O. SYMES

*From the Department of Surgical Science, University of Edinburgh*

Received for publication January 26, 1962

It was reported by Andreini, Drasher and Mitchison (1955) that enlargement of the spleen and lymph nodes occurred in C57BR mice which received transplants of an A-strain tumour, Sarcoma I, to which they were not susceptible. They attributed this to an immunological reaction, but did not offer any explanation of the fact that splenic and lymph node enlargement occurred also in susceptible (A-strain) mice which received transplants of the same tumour.

The present investigation began with the observation that splenic enlargement occurred regularly in A-strain female mice with spontaneous mammary cancer, and also in female A-strain and (A × ASW)$F_1$ hybrid mice bearing transplants of an A-strain mammary carcinoma, but not as a rule in mice of non-susceptible strains which received similar transplants. We have gone on to study the phenomenon in more detail, and in particular to determine firstly whether splenomegaly can be produced in susceptible mice with cell-free tumour textracts, and secondly, whether it occurs when the tumour is transplanted to animals which are normally non-susceptible if the resistance of the host is decreased by whole body irradiation.

### MATERIALS AND METHODS

### *Propagation of the tumour*

Female A-strain mice bearing spontaneous mammary carcinomata were killed by neck dislocation. The tumour was removed asceptically, and after all material which appeared to be necrotic had been discarded the remainder was cut into pieces (approximately 0·5 cm.$^3$) with scissors. One piece was transplanted subcutaneously by open operation under ether anaesthesia into each of a group of adult female A or (A × ASW)$F_1$ mice. The incision in the recipient was closed with a silk stitch.

Serial transplantation was carried out in the same manner every 14 to 21 days.

### *Preparation of tumour extracts*

Extracts were prepared by harvesting the viable tissue from a tumour, cutting it up finely in 5 ml. Hanks' solution, centrifuging at 200 g for five minutes and discarding the deposit. The dose per animal was 0·8 ml., i.e. about one sixth of the extract from one tumour, given by either subcutaneous or intraperitoneal injection.

### *Observations on animals bearing transplants*

The tumours were examined weekly by palpation and measured with a caliper in, and also perpendicular to, their long axis. The recipients were weighed weekly, and immediately before being killed, to the nearest 0·5 g.

Infection developed at the site of the graft in a few animals, and these were promptly killed and excluded from the experiment. Freedom from infection in the remaining mice was confirmed by histological examination of the tumour at the end of the experiment.

The spleen was removed immediately after death, weighed to the nearest 0·001 g., and then cut into two approximately equal pieces. One piece was used for histological study, some sections being stained with haematoxylin and eosin, some with Unna-Pappenheim stain, and some by silver impregnation using Marshall's (1956) modification of the Weil-Davenport method. The other piece was weighed and then gently broken up in Hanks' solution in a hand-operated glass homogenizer. The number of nucleated cells per cu.mm. in the resulting suspension was determined with a haemocytometer after dilution with 2 per cent acetic acid, and the total number of cells which would have been obtained from the whole spleen was estimated by calculating $CVM/m$ where

$C$ = haemocytometer count (cells/cu.mm.);
$V$ = volume of cell suspension;
$M$ = weight of whole spleen;
$m$ = weight of part of spleen used to prepare cell suspension.

*Irradiation*

The irradiation was given with a 230 kv Westinghouse machine (15 ma., 0·5 mm. Cu + 1 mm. Al, half-value layer 1·2 mm. Cu; focus-skin distance 50 cm.) under conditions of maximum back scatter. The dose rate was 149 r/min., measured in air at the surface of the animal nearest the tube.

### RESULTS

*A-strain mice developing spontaneous tumours*

Approximately 4 per cent of our A-strain female mice which are set aside for breeding develop spontaneous mammary cancer (Fig. 1).

The spleen weight and spleen ratio (i.e. 1000 × weight of spleen/weight of animal) for three animals bearing large spontaneous tumours are shown in the second category of Table I). Comparing these by Student's $t$ test with the corresponding values for normal A females, shown in the top category of Table I, we have for the absolute spleen weights, $t = 7{\cdot}56$, $n = 6$, $P < 0{\cdot}001$; and for the spleen ratios, $t = 3{\cdot}70$, $n = 6$, $P < 0{\cdot}02$. Both differences are thus significant at the conventional 5 per cent level despite the fact that in calculating the spleen ratios the weight of tumour-bearing animals includes the weight of the tumour.

Histologically the spleens of the tumour bearing animals showed changes of the kind associated with response to antigenic stimulation (Fig. 8). The Malpighian follicles were of normal size or slightly enlarged, and contained many activated lymphoid cells. The red pulp contained many pyroninophilic cells conforming to the description of immature plasma cells. There was no increase in the metalophil cell population and no evidence of extramedullary myelopoiesis.

*A-strain and A-hybrid mice receiving A-strain tumour transplants*

Transplants of A-strain mammary carcinomata (Fig. 2 and 3) to adult female A-strain or (A × ASW)$F_1$ mice, made by the technique described, were usually palpable after 5 days, and if undisturbed, killed the host after 35 to 50 days. Fourteen days after transplantation the mean diameter of the tumour was about 30 mm., and in animals killed at this stage, the absolute spleen weight, the spleen ratio, and the spleen nucleated cell count were all significantly increased (Table I). The weight of the liver remained within normal limits.

Details of the statistical comparisons with the corresponding control values are as follows :

*For A mice*

Absolute spleen weight : $t = 12{\cdot}8$, $n = 13$, $P < 0{\cdot}001$.

Spleen ratio : $t = 3{\cdot}62$, $n = 13$, $P < 0{\cdot}01$.

Spleen count : $t = 3{\cdot}87$, $n = 8$, $P < 0{\cdot}01$.

*For* $(A \times ASW)F_1$ *mice*

Absolute spleen weight : $t = 3{\cdot}00$, $n = 12$, $P < 0{\cdot}02$.

Spleen ratio : $t = 2{\cdot}80$, $n = 12$, $P < 0{\cdot}02$.

Spleen count : Difference striking but not statistically significant, possibly owing to small number of observations.

On the other hand, in mice which had received a subcutaneous or intraperitoneal injection of cell-free tumour extract 14 days previously the absolute spleen weight, spleen ratio, and spleen nucleated cell count did not differ significantly from the values found in the controls.

Tumour bearing animals showed usually a slight but progressive fall in blood haemoglobin level (Table II), but this never appeared to be sufficient to account

TABLE II.—*Changes in the Peripheral Blood of Female A-strain Mice Bearing Mammary Carcinoma Transplants*

| Weight of mouse on Day 14 | Haemoglobin (per cent) | | | Polymorphonuclear count (cells/cu.mm.) | | | Lymphocyte count (cells/cu.mm.) | | | Spleen ratio on Day 14 |
|---|---|---|---|---|---|---|---|---|---|---|
| | Day 0 | Day 7 | Day 14 | Day 0 | Day 7 | Day 14 | Day 0 | Day 7 | Day 14 | |
| 23·5 | 104 | 94 | 58 | 4460 | 2380 | 5730 | 3800 | 3430 | 6470 | 7·23 |
| 21·5 | 98 | 90 | 79 | 2810 | 940 | 3020 | 2390 | 1360 | 2280 | 7·96 |
| 20·5 | 104 | 93 | 67 | 3980 | 3150 | 4160 | 3820 | 3550 | 3840 | 7·80 |
| 22·5 | 96 | 96 | 79 | 1830 | 3010 | 3650 | 1830 | 3390 | 4850 | 8·89 |
| 21·5 | 102 | 89 | 49 | 3070 | 2340 | 10,950 | 2230 | 2860 | 4050 | 8·37 |
| 20·5 | 83 | 72 | 88 | 920 | 2060 | 3940 | 3680 | 6540 | 3360 | 8·00 |
| 19·5 | 92 | 90 | 82 | 2900 | 2290 | 6160 | 3700 | 2810 | 5040 | 13·95 |
| 18·0 | 78 | 90 | 87 | 1790 | 1130 | 5920 | 3810 | 2630 | 8880 | 8·94 |
| 20·0 | 75 | 91 | 62 | 1380 | 1530 | 5060 | 2670 | 3570 | 4140 | 11·30 |
| 18·0 | 90 | 85 | 79 | 1870 | 3160 | 4970 | 2030 | 3040 | 2030 | 13·00 |

Day 0 denotes the day of transplantation.

for the splenomegaly. The absolute polymorphonuclear and lymphocyte counts sometimes showed a temporary fall, but by the fourteenth day were usually moderately increased in comparison with the value in the same animal before transplantation (Table II). The mean number of nucleated cells obtained from the marrow of one femur 14 days after transplantation was 63 per cent of that obtained from control mice (Table III), and the difference is significant at the 2 per cent level ($t = 3{\cdot}00$, $n = 8$, $P < 0{\cdot}02$).

TABLE III.—*Nucleated Marrow Cell Counts from One Femur in Female A Strain Mice Bearing Mammary Carcinoma Transplants and Normal Mice*

| Category | Weight of mouse (g.) | Spleen ratio | Marrow cell count (1 femur) (millions) | Mean marrow cell count (millions) |
|---|---|---|---|---|
| Normal mice . . . . . | 21·0 | 5·33 | 12·0 | 11·8 |
| | 22·5 | 5·20 | 14·0 | |
| | 23·0 | 4·48 | 13·5 | |
| | 22·0 | 5·10 | 11·5 | |
| | 21·0 | 7·05 | 7·8 | |
| Mice which had received tumour transplants 2 weeks previously | 20·5 | 8·00 | 6·5 | 7·4 |
| | 19·5 | 13·95 | 5·0 | |
| | 18·0 | 8·94 | 8·7 | |
| | 20·0 | 11·30 | 10·4 | |
| | 18·0 | 13·00 | 6·5 | |

Histologically the spleens of the tumour-bearing animals showed changes similar to those seen in the mice with spontaneous tumours but more intense (Fig. 6). In addition in some sections the number of polymorphonuclear leucocytes in the red pulp was a little greater than normal.

Cell suspensions prepared from spleens of normal and tumour bearing mice were injected into newborn or five-day-old isogenic recipients to find out whether the factor responsible for the splenomegaly in tumour-bearing animals could be passaged. The results, with appropriate controls, are shown in Table IV. It will be seen by comparing the appropriate lines in the table that passaging did not occur.

---

### EXPLANATION OF PLATES

FIG. 1.—Spontaneous mammary carcinoma in A-strain female mouse. Note the well marked acinar arrangement of the cells. H. and E. ×340.

FIG. 2.—Intra-strain transplant of mammary carcinoma after 2 weeks. This tumour has retained its well differentiated appearance. H. and E. ×340.

FIG. 3.—Intra-strain transplant of mammary carcinoma after 2 weeks. Here the tumour shows almost complete lack of differentiation and consists mainly of spindle-shaped cells. H. and E. ×340.

FIG. 4.—On the left are six spleens from ASW mice. The top three are from normal mice, the next is from a mouse which received 350 r irradiation and a transplant of A mammary carcinoma 2 weeks previously, and the two lowest are from mice which received 550 r irradiation and transplants of A mammary carcinoma 2 weeks previously. The corresponding tumours are shown on the right.

FIG. 5.—Spleen of normal A-strain female mouse, to show size of follicles and degree of cellularity of the red pulp. H. and E. ×150.

FIG. 6.—Spleen of A-strain female mouse bearing transplanted A-strain mammary carcinoma for 2 weeks showing intense accumulation of plasma cells and their precursors in the red pulp. H. and E. ×150.

FIG. 7.—High power view of splenic red pulp from normal A-strain female mouse. Pyronin methyl-green ×600.

FIG. 8.—High power view of splenic red pulp from A-strain female mouse bearing spontaneous mammary carcinoma showing many immature and mature plasma cells. Pyronin methyl-green ×600.

FIG. 9.—Spleen of normal ASW female mouse to show size of follicles and degree of cellularity of red pulp. H. and E. ×150.

FIG. 10.—Spleen of ASW female mouse, following 550 r whole body irradiation and transplantation of A-strain mammary carcinoma. The follicles are reduced in size and the red pulp contains numerous darkly staining plasma cells and their precursors. H. and E. ×150.

Woodruff and Symes.

*ASW and CBA mice receiving A-strain tumour transplants*

Transplants of A-strain mammary carcinomata to untreated ASW and CBA mice did not increase in size and by 14 days were almost completely necrotic. Some ASW mice which received transplants were kept for 3 months but there was never any reappearance of the tumour. Mice killed 2 weeks after transplantation showed no increase in either the absolute or relative spleen weights (Table V), and little or no change in histological appearance. On the other hand, a greatly enlarged lymph node (mean weight 22 mg.) was found regularly in the ipsilateral axilla. Histological examination showed complete loss of the normal follicular architecture of the node associated with local accumulations of plasma cells, many of which appeared to be immature.

In ASW mice which received 550 r whole body irradiation before transplantation, grafts of tumours which were rapidly destroyed in normal ASW mice without causing splenomegaly grew progressively for a week or more and still contained viable looking tissue on histological examination 14 days after transplantation. As will be seen from Tables V and VI, the absolute and relative spleen weights were all greater than in normal ASW mice, and *a fortiori* greater than in irradiated controls which did not receive grafts (Fig. 4). As in the case of A-strain tumour-bearing mice the weight of the liver remained within normal limits.

Details of the statistical comparison between irradiated tumour-bearing ASW mice and normal ASW mice are as follows :

Absolute spleen weights : $t = 7 \cdot 33$, $n = 27$, $P < 0 \cdot 001$.
Spleen ratios : $t = 17 \cdot 2$, $n = 27$, $P < 0 \cdot 001$.

Each difference is thus highly significant. On the other hand there is no significant difference in the cell counts despite the fact that the number of observations is quite large.

Histologically the spleens of the tumour-bearing irradiated animals showed accumulation of pyroninophilic cells in the pulp as in tumour-bearing A-strain mice, but the plasma cells were in the main more mature. The Malpighian follicles, on the other hand, were smaller than normal (Fig. 10), and we attribute this fact, and also the absence of increase in the total nucleated spleen cell count, to cellular damage caused by the irradiation.

Irradiation in a dosage of 350 r was tested with two tumours. It had no apparent effect with one, but some effect with the other.

The effect of 550 r whole body irradiation on tumour survival, and also on the absolute and relative spleen weights in tumour recipients 14 days after transplantation, was very greatly reduced if the recipients were given in addition an intravenous injection of 50 million spleen cells from an ASW mouse which had been immunized with a transplant of the same tumour 15 days previously (Table VI).

### DISCUSSION

In these experiments an increase in absolute and relative spleen weight, associated with histological changes in the spleen characteristic of antigenic stimulation, was regularly observed in mice in which a tumour grew progressively, at least for a few days. This occurred (1) with spontaneous tumours, (2) with tumours transplanted within the strain of origin or to $F_1$ hybrids of this and another inbred strain, and (3) with tumours transplanted to irradiated (550 r) mice of a nonsusceptible strain.

On the other hand, when a tumour was transplanted to a mouse in which it did not appear to grow progressively even temporarily, while there was regional lymph node enlargement, the absolute and relative spleen weights, and the splenic nucleated cell count, remained within normal limits. This occurred when the recipient was an untreated mouse of a non-susceptible strain, or an irradiated member of such a strain which had been re-equipped with spleen cells from an isogenic animal which had rejected a graft of the same tumour.

Two explanations may be suggested for the correlation between progressive tumour growth and splenomegaly.

The first is that the changes observed in the spleen were a manifestation of a reaction to damage caused by the tumour. The mild anaemia which was observed in some of the tumour-bearing animals did not seem to provide a sufficient explanation, but other forms of damage of a less obvious kind cannot be entirely excluded.

The second explanation, which accords well with the histological findings, is that the splenic changes were a manifestation of an immunological reaction evoked by antigens liberated from the tumour. This would imply firstly that the tumours under investigation possessed at least one antigen not represented in the normal tissues of the strain of origin, and secondly that in non-susceptible animals the local reaction and the reaction in the regional lymph nodes was so effective that the tumour was destroyed before the spleen was exposed to sufficient antigenic stimulation to show morphological evidence of immunological activity.

The hypothesis that a tumour may differ immunologically from the host in which it originates, not merely in the sense of lacking some of the antigens present in normal tissues but in possessing antigens which normal tissues lack, has important implications because, if true, it provides a rational basis for attempts to forge immunological weapons for use against cancer. It dates from the work of Lumsden in the period between the two world wars (for review see Woodruff, 1960). Lumsden's interpretation of his results has been severely criticised but the concept of tumour specific antigens has been put forward again more recently by Kidd (1946), Gorer and Amos (1956), and others (see Snell, 1958).

The subsidiary hypothesis that a chronic antigenic stimulus is needed to produce gross changes in the spleen appears reasonable, but there do not appear to be any reported transplantation experiments which tell decisively for or against it.

It is possible to construct a variant of each of the explanations considered above, and attribute the splenomegaly in the tumour-susceptible animals to injury or antigenic stimulation caused by a tumour virus. The low incidence of mammary carcinoma in our A-strain mice makes it extremely unlikely that they carry the Bittner factor, however, and the failure to produce splenomegaly in adults with cell-free tumour extracts, or in immature animals with splenic cells from tumour-bearing adults, also tells strongly against a virus as the causal agent.

The consistency of the splenomegaly despite the precautions taken to exclude infected tumours, and the absence of any mortality on transplantation of the tumour into irradiated ASW mice, would seem to exclude reaction to bacterial infection as an explanation of the results.

SUMMARY

Experiments are described in which an increase in absolute and relative spleen weight, associated with histological changes characteristic of antigenic stimulation, occurred in A-strain mice bearing spontaneous or transplanted A-strain mammary carcinoma. Similar changes occurred in irradiated, but not in normal, ASW mice which received grafts of the same tumour.

It is suggested that the splenic changes were due to an immunological reaction evoked by antigen liberated from the tumour. This would imply firstly that the tumour possessed one or more antigens not present in normal A-strain tissues, and secondly that the antigenic stimulus was insufficient to cause splenomegaly unless the tumour grew progressively, at least for a time, after transplantation.

This work was supported by a generous grant from the British Empire Cancer Campaign, of which grateful acknowledgement is made. One of us. (M. O. S.) is a Medical Research Council Scholar and is indebted to the Council for this support.

We are grateful to Dr. Angus Stuart for guidance in histological matters and to Dr. N. A. Mitchison for reading the manuscript. Finally we wish to acknowledge the expert technical help of Mr. N. Samuel and Mr. G. Aitchison.

### REFERENCES

Andreini, P., Drasher, M. L. and Mitchison, N. A.—(1955) *J. exp. Med.*, **102**, 199.
Gorer, P. A. and Amos, D. B.—(1956) *Cancer Res.*, **16**, 338.
Kidd, J. G.—(1946) *J. exp. Med.*, **83**, 227.
Marshall, A. H. E.—(1956) ' An Outline of the Cytology and Pathology of the Reticular Tissue '. Edinburgh (Oliver and Boyd).
Snell, G. D.—(1958) In : ' Physiopathology of Cancer ", edited by F. Homberger. London (Cassel), pp. 313–335.
Woodruff, M. F. A.—(1960) ' The Transplantation of Tissues and Organs ". Springfield, Ill. (Thomas), p. 148.